# Effectiveness of a Worksite-Based Lifestyle Intervention on Employees’ Obesity Control and Prevention in China: A Group Randomized Experimental Study

**DOI:** 10.3390/ijerph19116738

**Published:** 2022-05-31

**Authors:** Jingxia Kong, Ying Chen, Yingjing Zheng, Lin Zhu, Boyan Chen, Xiao Cheng, Mengna Song, Donald L. Patrick, Shirley A. A. Beresford, Hongmei Wang

**Affiliations:** 1 Department of Investment and Insurance, Zhejiang Financial College, Hangzhou 310018, China; kongjx_79@126.com; 2 Department of Social Medicine, Zhejiang University School of Public Health, Hangzhou 310058, China; chenying1996@zju.edu.cn (Y.C.); zhengyingjing@zju.edu.cn (Y.Z.); chen_b_y@zju.edu.cn (B.C.); xcheng92@163.com (X.C.); 21618445@zju.edu.cn (M.S.); 3 Shanghai Health Development Research Centre (Shanghai Medical Information Centre), Shanghai 200031, China; zhulin@shdrc.org; 4 Department of Health Systems and Population Health, University of Washington, Seattle, WA 98195, USA; donald@uw.edu; 5 Department of Epidemiology, University of Washington, Seattle, WA 98195, USA; beresfrd@uw.edu

**Keywords:** worksite, obesity, lifestyle intervention, dietary, physical activity

## Abstract

**Background**: This study was to culturally adapt a lifestyle intervention for employees’ obesity control and prevention using a participatory process, and evaluate the effectiveness of the project at worksites. **Methods**: A group randomized experimental study included four worksites (two intervention, two control) in the Yangtze River Delta in China was conducted. A total of 388 participants (216 in the intervention worksites and 172 in the control worksites) were finally recruited from 955 employees at the four worksites (464 in the intervention worksites and 491 in the control worksites). The final evaluation was completed by two hundred and seventy-eight employees (159 in the intervention worksites and 119 in the control worksites, respectively). Data of demographic information, weight, BMI, waist circumference, hip circumference and weight-related behaviors including diary behaviors and physical activities were collected before and after a 12-month intervention and analyzed using descriptive statistics, *t*-test, chi-square test, linear mixed regression and logistic mixed regression. **Results**: Although the intervention worksites had a reduction in body mass index (23.21 to 22.95, *p* < 0.01), hip circumference (95.97 to 95.28, *p* = 0.03) and waist-to-height ratio (0.49 to 0.48, *p* = 0.01), the differential changes compared to those of the control group were not statistically significant. The frequency of sweet beverages (−1.81, 95%CI: −0.52, −3.11), frequency of vegetable intake (5.66, 95%CI: 1.59, 9.74), daily servings of vegetables (0.53, 95%CI: 0.24, 0.82), frequency of fruit intake (3.68, 95%CI: 1.25, 6.12), daily servings of fruit (0.26, 95%CI: 0.44, 0.92), daily servings of vegetables and fruit (0.79, 95%CI: 0.43, 1.16), daily steps (863.19, 95%CI: 161.42, 1564.97) and self-efficacy to change physical activity (OR = 1.91, 95%CI: 1.02,3.60) were more improved in the intervention group than were those measures in the control group. **Conclusions**: The worksite-based lifestyle intervention project for obesity control and prevention improved several employees’ dietary behaviors and physical activities at worksites in China in a short time. Long-term intervention with larger samples in more worksites should be further examined.

## 1. Introduction

The prevalence of overweight and obesity in China has increased rapidly since the 1980s [1]. According to the China National Nutrition Surveys (CNNSs), the overweight and obesity rate in adults increased, respectively, from 5.4% and 0.1% in 1982 to 34.3% and 16.4% in 2015–2019 [2,3,4]. The obesity rate of employees, who consist mainly of a working population, has, accordingly, experienced a rapid increase and has been highlighted as a major public health concern [5]. Evidence has indicated that obesity is an independent risk factor for an expanding set of chronic diseases, including cardiovascular disease [6,7,8], diabetes mellitus [9,10,11], stroke [12,13] and many cancers [14,15,16,17]. In consequence, it is associated with an increased risk of morbidity and reduced life expectancy [18,19] and, therefore, is correlated with increased healthcare and medical costs [20]. In China, the contribution of overweight and obesity to deaths associated with non-communicable diseases (NCDs) was 11.1% in 2019, increased from 5.7% in 1990 [1]. While, for employees, apart from personal consequences including health damage and economic burden, obesity has socio-economic consequences including low productivity [21], absenteeism from work, sick leave, disability, and injuries [22]. Accordingly, employers also have an incentive to prevent and control employees’ obesity [23].

Obesity is mainly caused by an imbalance between energy intake and consumption, because of individuals only experiencing obesity for about 40 years. With rapid economic development, lifestyle factors such as diet and physical activity have changed a lot in the past few decades in China, which are highly relevant to obesity [24,25,26]. The traditional diet with cereals and vegetables has gradually transitioned to a western-style diet with high-sugar and high-fat [27]. National data on dietary intakes showed an increase in the energy intake percentage from fat from 18.4% to 31.5% between 1982 and 2012 in China [4,28]. In addition, at the same time, physical activity has decreased due to automated work, inactive leisure activities and motorized transport [29,30]. Evidence has showed that physical activity levels in adults declined from 427.8 metabolic equivalent of task (MET)-h per week in 1991 to 246.0 MET-h per week in 2011 [29], which was indicated as a risk of obesity [31,32]. Therefore, both controlling dietary intake and increasing physical activity have been consistently highlighted as crucial factors in preventing weight gain [33,34].

Worksites are an appropriate setting for obesity prevention interventions [35]. Employees spend a substantial period of time at work [36], and worksites have a number of characteristics, such as established channels of communication and social support networks, that could support multi-component, ecological and individual interventions [37]. The efficacy of worksite-based interventions on obesity [38,39,40] has been reported in a number of studies and accepted as an ideal pattern internationally in recent years [37].

In terms of intervention design, evidence has suggested that health-related behavioral interventions based on certain behavioral change theories are much more effective than those that lack theoretical guidance [41,42]. The social-cognitive theory (SCT) [43] is a theory emphasizing person, environment and their interaction, which has been proven to be efficacious in the implementation of weight-control interventions [44,45]. Community-based participatory research is an approach based on the equal participation of community members and researchers [46]. Community members participate in the design and implementation of interventions suitable for their needs [47]. It has been proven to enhance the effectiveness of interventions and save time and cost [48].

However, as far as we are aware, few systematic worksite-based interventions on obesity control and prevention guided by a particular health-behavior-change theory have been carried out in China. This study aimed to adapt a health-behavior intervention project based on SCT at worksites [49] for preventing employees’ weight gain, and then assess the effectiveness using a group randomized experimental study. The potential of the work reported here is that many parts of China have not experienced the obesogenic environment for very long, and so may be at an earlier stage in the obesity epidemic [1,2,3,4]. There may be a greater chance of identifying efficacious interventions targeting diet and exercise.

## 2. Methods

This was a group randomized controlled trial conducted in four worksites in Yangtze River Delta in China. This study was approved by Zhejiang University School of Medicine Ethics Committee and was registered with the Chinese Registry of Clinical Trial (IRCTID: ChiCTR-IOC-17011256).

### 2.1. Study Design and Subjects 

This study was conducted between 2017 and 2018, in which worksites were the unit of randomization and intervention. Four worksites in Yangtze River Delta in China were recruited for the study and randomly assigned to intervention or control conditions. Data were collected at baseline and after a 12 months intervention. Written informed consent was obtained from participants before enrollment in the study. The control group did not receive any intervention measures except for the feedback of the baseline and follow-up measurements. We provided the option of delayed intervention although this was not applied to the control group until after the study.

### 2.2. Worksites

Study recruitment advertisement was sent to the worksites with a reported company size of 50 to 150 employees in Yangtze River Delta in China using email, WeChat and telephone. Interested worksites were interviewed to confirm eligibility, and those which had a large proportion (>50%) of desk-based employees, had been operating for more than three years, and had not ever hosted a health management program were eligible. Four worksites were finally included in the study, which were paired according to the demographic and working characteristics and then randomly assigned into intervention worksites (*n* = 2) and control (*n* = 2) worksites.

### 2.3. Participants

All employees from four worksites were invited to participate between January 2017 and September 2017. Employees were considered eligible if they were full-time employees aged ≥18 years old, had not received clinical weight-loss treatment, had signed informed consent, and were not pregnant at the time of recruitment. The calculation of the initial sample size was based on the difference in BMI of 0.4 reported in the literature [46,50]. Assuming an intraclass correlation coefficient (ICC) of 0.01, a two-tailed power of 80% and a 0.05 alpha level, it was calculated that 2 clusters with 70 participants in each cluster would be needed in each group to detect the difference. Allowing for 20% drop-out rate, a total of 179 patients from 2 worksites in each group needed to be included. A total of 388 participants (216 in the intervention worksites and 172 in the control worksites) were finally recruited from four worksites, with 955 employees totally at the four worksites (464 in the intervention worksites and 491 in the control worksites). Participants completed questionnaires of demographic information and weight-related life behaviors including diary behaviors and physical activities, and weight-related indicators including measured weight and height, waist circumference, and hip circumference at baseline from March 2017 to October 2017. After a 12-month intervention, the same questionnaires and measurement were conducted again in December 2018.

### 2.4. Randomization and Blinding

After baseline measurements, worksites were paired according to the characteristics mainly including the scale of the enterprise; the nature of the enterprise (state-owned or private), which could be obtained through public information; and the demographic characteristics of employees including gender, age, etc. which could be provided by the human resources departments, and randomly assigned into intervention worksites (*n* = 2) and control (*n* = 2) worksites by drawing lots.

Blinding of the participants and the intervention implementers was not possible because of salient differences in procedures between intervention worksites and control worksites. Our data collectors and analysts were blind to group assignment.

### 2.5. Measures

#### 2.5.1. Control Worksites

The control worksites received both baseline measurement and final measurement. The results were sent back to the worksites together with the health education tips after the study.

#### 2.5.2. Intervention Worksites

The intervention worksites received a 12-month community-based participation intervention, which is a cooperative process that engages community (worksite) members and researchers as equals [46]. The intervention, based on SCT, mainly including individual level, environmental level and organizational level, was implemented from December 2017 to November 2018.

(1) Individual level

The goals at this level were to improve the employees’ health awareness, compliance and skills to increase their healthy food consumption and physical activities.

- Six lectures on weight management, healthy diets, physical activities and health preservation of traditional Chinese Medicine were given once every two months from January 2018 to November 2018.
- Health information about weight-related lifestyle changes was pushed by the WeChat Subscription—“Official Health Management” 2–3 times weekly, as well as the enquiry of dietary energy and energy consumption of various sports being provided throughout the intervention period. This was also available to all employees in the intervention worksite.
- Several diet and exercise teams were set up voluntarily based on employees’ interests and personal relationships to monitor and improve their daily behaviors by recording the daily diet or exercise and remind each other. Team members were asked to record their daily diet or exercise. In addition, members of the diet teams supervised what each other ate and drank during meals and snacks, and the person with unhealthy food would be reminded by others on the spot. Members of the exercise teams went to exercise regularly at the agreed time and place. For example, the members of the walking team reminded each other after lunch every day and walked together along the set route.
- Walking routes and activities suitable for the workplaces were designed and opened to all employees in each worksite to improve employees’ access to physical activities.
- Automatic ranking of daily steps was provided by the WeChat Subscription of this study and material rewards were provided by the worksites to enhance employees’ enthusiasm to participate in the activities.

(2) Environmental level

The goals at this level were to create a positive environment at workplaces, and increase the employees’ access to healthy diet and physical activities.

- A kick-off event was held at each worksite to introduce the program to the employees.
- Posters about health, diets, and physical activities were displayed on the bulletin board at the worksites.
- Food models and scales were placed at the gate, restroom or conference room.
- Signs encouraging walking were posted beside elevators and stairs at workplaces.
- A fitness area was set up at each worksite for the employees where some sport equipment was provided freely.
- The environment of dining area and snack bar of the worksites was improved. The food calories were displayed in the dining area. The supplies of healthy food were increased including fruit, milk and waters while the supplies of high-calorie food such as instant noodles, french fries and Coca Cola were reduced in the snack bar of worksites. A certain discount was also encouraged to offer to staff on healthy food.
- The “Award for healthy diet showing” and “Exercise challenge” activities were launched for all employees at worksites based on WeChat Subscription to facilitate healthy diet and physical activities maintenance.

(3) Organizational level

- An employee advisory board (EAB), which consisted of four to seven employees from all occupational sectors in the worksite, was established in each worksite and worked closely with research team to design and implement intervention activities [51].
- Positive policies were created in worksites including food subsidy policies for snack bars, financial policies for clubs and groups of dietary and activities and reward policies for “Exercise challenge” and “Show healthy diet and win prizes”.

In terms of participants’ compliance, the literature shows that the compliance of diet intervention and exercise intervention is basically maintained at about 50%, and the low is even only about 10% [52]. Therefore, we set a minimum expected engagement level of 30% in the methods according to the literature.

### 2.6. Outcomes

(1) Primary outcomes

The primary outcomes were weight-related indicators including measured weight, body mass index (BMI), waist circumference (WC), hip circumference (HC), waist-to-hip ratio (WHR) and waist-to-height ratio (WHtR). Although the gold-standard DEXA approach would allow a more detailed assessment of body-composition changes, limited by funding and employees’ engagement willingness, we chose indicators that are easier to collect, such as BMI, waist circumference and hip circumference instead of DEXA approach. Weight and height were measured by trained study personnel with the participants standing without shoes and heavy outer garments using a calibrated mechanical scale and stadiometer (model RGZ-120), to the nearest 0.1 kg and 0.1 cm, respectively. Both weight and height were assessed twice, and the average of the two measurements was recorded. BMI was calculated as weight (kg) divided by the square of height (m^2^) using physically measured data for these analyses. WC and HC were measured twice with a tape after taking off the coat. The average of the two measurements was recorded, to the nearest 0.1 cm. WHR and WHtR were calculated from these two averages.

(2) Secondary outcomes

The secondary outcomes included changes in weight-related lifestyle behaviors including dietary behaviors and physical activities.

- Assessment of Dietary Behavior

Studies have demonstrated that certain dietary choices and eating behaviors are strongly related to body weight, including fried-food intake [53,54], sweetened-beverage and food consumption [55,56], fruit and vegetable intake [57], fast-food meals [58,59,60], frequency of eating out [61,62], task eating (eating while doing other activities) [36], and self-efficacy to change dietary behaviors [59,63]. Therefore, the monthly servings of fried food, sweetened beverages and snacks were assessed by self-reported frequency (① Never; ② Less than once per month; ③ 1–3 times per month; ④ 1–2 times per week; ⑤ 3–4 times per week; ⑥ 5–6 times per week; ⑦ once per day; ⑧ 2 times per day; ⑨ 3 times per day) and servings per time (① ≤1/2; ② 1; ③ 2; ④ 3; ⑤ ≥4; ⑥ None of above). Frequency of fast-food meals and eating out were self-reported via a question: “How many times in a week or month do you have a dinner in a place such as McDonald’s^®^, Burger King^®^, Kentucky Fried Chicken^®^, or eating out with your friends or colleagues?” Responses were given as number of times per week or number of times per month. All responses were converted to number of times per week. Task eating was assessed via a single item: “How often do you eat food (meals or snacks) while doing another activity—for example, watching TV, working at a computer, reading, or playing video games?”. Response options were presented on a 5-point Likert scale ranging from 1 (“never”) to 5 (“always”). Self-efficacy to change dietary behavior was assessed via a single item: “How sure are you that you can stick to monitor your diet as planned in your daily life?” Responses were on a 5-point Likert scale ranging from 1 (“extremely sure”) to 5 (“not sure”). “Extremely sure”, “very sure” and “relatively sure” were considered as “high self-efficacy”; “a little sure” and “not sure” were considered as “low self-efficacy”.

- Assessment of Physical Activities

Based on previous research, free-time physical activity of at least 10 min, frequency of walking weekly, daily steps, and self-efficacy were used as indicators to evaluate physical activities [53,64]. Free-time physical activity of at least 10 min was assessed using a modification of the Godin–Shephard Leisure-Time Physical Activity Questionnaire (GSLTPAQ) [65], which is both reliable with a reported test–retest correlation coefficient ranging from 0.48 for light activity to 0.94 for strenuous activity and valid in relation to maximal oxygen consumption [65]. It is widely used in measurement of physical activity in clinical and epidemiological studies [66,67,68]. The questionnaire provides direct estimates of frequency of vigorous, moderate, and light exercise, and assessment of frequency of sweat-inducing exercise. Physical activity scores (Godin index) were calculated according to the recommended protocol [69], which uses metabolic equivalent task (MET) units. Higher scores indicated more activity. Frequency of walking weekly was measured used a single-item, adapted from the International Physical Activity Questionnaire (IPAQ): “During the last 7 days, how many days did you walk for at least 10 min at a time?” Daily steps were measured used pedometers (Omron HJ-321) for 7 days. Self-efficacy to change physical-activity behavior was assessed via a single item: “How sure are you that you can increase your level of physical activity on a regular basis?” Responses were on a 5-point Likert scale ranging from 1 (“extremely sure”) to 5 (“not sure”). “Extremely sure”, “very sure” and “relatively sure” were considered as “high self-efficacy”; “a little sure” and “not sure” were considered as “low self-efficacy”.

### 2.7. Data Collection

At the baseline, general demographic and weight-related lifestyle behaviors including age, gender, marital status, educational level, household income, daily servings of fruits and vegetables, sweated beverage and fried food, the frequency of eating at fast-food restaurants, eating out and eating while doing another activity such as watching TV, the frequency of walking weekly, leisure-time exercise and self-efficacy were collected by self-administered questionnaires. Daily steps for 7 days were recorded by research assistants on site. Height, weight, WC and HC were measured by the research assistants at worksites. All the surveys were repeated after 12 months.

### 2.8. Statistical Analysis 

Baseline general characteristics of age were described using means and SDs. The categorical variables in the baseline demographic characteristics, including gender, marital status, educational level and household income, were described using frequency and constituent ratios. Independent sample *t*-tests for continuous variables and chi-square tests for categorical variables were adopted to examine differences in the demographic characteristics between the intervention worksites and the control worksites at baseline. Paired *t*-tests for continuous data and chi-square test for categorical data were used to determine within-group differences between baseline and follow-up. The main analyses used linear mixed regression to compare the differences in changes in continuous data from baseline to follow-up between the intervention worksites and the control worksites. All models were adjusted for the effects of demographic characteristics including age, gender, education level, household income and marital status, with the clusters of worksites being included as random effects, and the effects of time and the intervention included as fixed effects. Similarly, for the main analyses of binary outcomes, logistic mixed regression was used to analyze the associations between weight-related behaviors and independent variables. Independent variables included group (control/intervention), the clusters of worksites, weight-related behaviors at baseline and individual characteristics including age, gender, educational level, household income and marital status, respectively. Again, the clusters of worksites were fitted as random effects. The effects of weight-related behaviors at baseline and individual characteristics including age, gender, educational level, household income and marital status were included as fixed effects, respectively, in the models. Odds ratio (or) and 95% confidence interval (CI) were used to express the correlation level between dependent variables and independent variables. SPSS 28 for Windows (IBM, Armonk, NY, USA) was adopted for our data analysis.

## 3. Results

**Figure 1:**
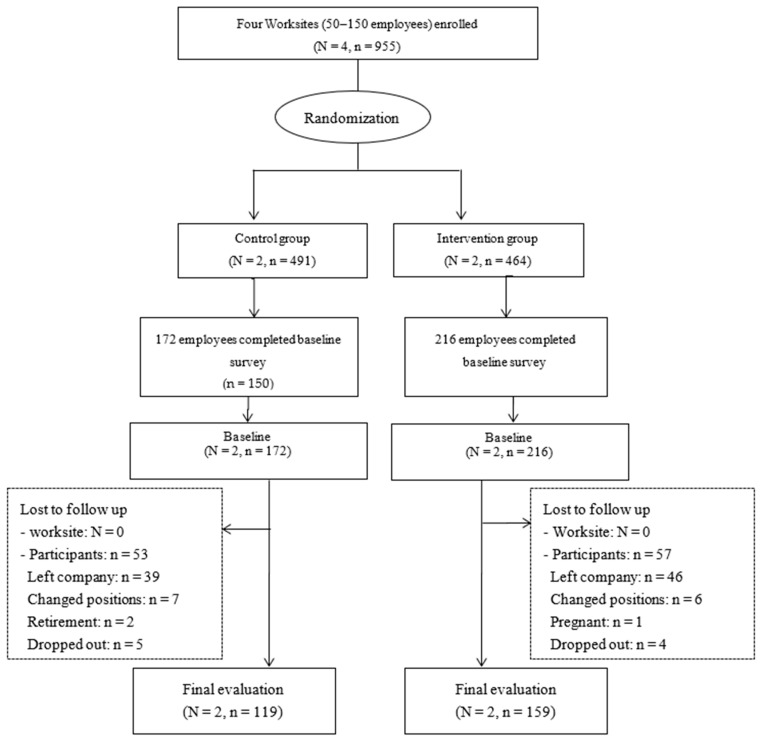
Flow of site and participant recruitment, screening, and assessment.

A total of 955 employees in four worksites (464 in the intervention worksites and 491 in the control worksites) were invited to participate in the study. A total of 388 (216 in the intervention worksites and 172 in the control worksites) completed the baseline evaluation and 278 (159 in the intervention worksites and 119 in the control worksites) completed the final evaluation after follow up. The response rate was 40.63% (46.6% in the intervention worksites and 35% in the control worksites). No significant difference was observed in the response rate between the two groups. Intention-to-treat analysis (ITT) was preformed and reported in major outcomes. Figure 1 shows the participant flow chart in the study.

Baseline socio-demographic characteristics were comparable for intervention and control worksites except for the age and the educational level (Table 1).

(1) Weight-related indicators

As shown in Table 2, the intervention worksites had a reduction in BMI (23.21 to 22.95, *p* < 0.01), HC (95.97 cm to 95.28 cm, *p* = 0.03) and WHtR (0.49 to 0.48, *p* = 0.01) between baseline and follow up, whereas there was no statistical difference in these three indicators in the control group. The between-groups effects were estimated using linear mixed regression. When the effect of group was adjusted for the worksites and the demographic characteristics including age, gender, marital status, educational level and household income, no statistically significant between-group intervention effects were observed on weight-related indicators.

(2) Dietary behaviors

As shown in Table 3, the intervention worksites had an increase in fast-food frequency (1.12 to 2.08, *p* < 0.01). There was no statistical difference in other indicators. The control group had an increase in vegetable-intake frequency (34.66 to 41.22, *p* < 0.01) and fast-food frequency (1.30 to 2.09, *p* < 0.01). No difference was observed in other indicators. 

Meanwhile, when the effect of group was adjusted for the demographic characteristics and the worksites using a linear mixed model, significant between-group intervention effects were observed in frequency of sweet beverage (−1.81, 95%CI: −0.52, −3.11), frequency of fruit intake (3.68, 95%CI: 1.25, 6.12), daily servings of fruit (0.26, 95%CI: 0.44, 0.92), frequency of vegetable intake (5.66, 95%CI: 1.59, 9.74), daily servings of vegetables (0.53, 95%CI: 0.24, 0.82), and the daily servings of vegetables and fruit (0.79, 95%CI: 0.43, 1.16). No statistical difference was observed in other indicators.

(3) Physical activities

As shown in Table 4, after a 12-month intervention, the intervention worksites had a significant increase in the proportion of exercise long enough to sweat (53.0% to 63.6%, *p* = 0.02). No statistical difference was observed in other indicators of physical activities. In the control group, there was a significant decrease in daily steps (6986.14 to 4994.83, *p* < 0.01) and an increase in the proportion of exercise to sweating (54.3% to 71.4%, *p* = 0.03). No statistical difference was observed in other indicators.

Meanwhile, when the effect of group was adjusted for the demographic characteristics and the worksites using linear mixed regression, significant between-group intervention effect was observed in daily steps (863.19, 95%CI: 161.42, 1564.97). Logistic mixed regression showed that the employees in the intervention worksites were more likely to have high exercise self-efficacy (OR = 1.91, 95%CI: 1.02, 3.60).

## 4. Discussion

This was a group randomized experimental study with a participatory process that found improvements in weight-related behaviors such as vegetables/fruits intake, frequency of sweetened beverages, daily steps and self-efficacy to change physical-activity behavior after the 12-month intervention. 

Although the BMI, HC and WHtR of the intervention worksites were significantly improved between baseline and follow-up, no statistical difference was observed compared with the change in control worksites using the linear mixed model. An explanation for our finding in contrast to some other studies might be that participation in this study was not restricted to employees who were overweight or obese. The intervention targeted the general population—both those who were currently overweight or obese and those with normal weight. Prior studies also showed that BMI and waist circumference do not decrease significantly after 1 year of intervention when all employees, rather than only those overweight or obese at worksites, are included [70,71,72], which implies the difficulty of weight loss for general populations at workplaces. The average weight gain among subjects (20 to 40 years old) in the population is about 1.8 to 2.0 pounds per year [73]. Therefore, in order to prevent weight gain, energy expenditure should be increased by 100 kcal per day through walking an extra mile (2000 to 2500 extra steps) each day, or energy intake can be decreased by reducing the supply of food at each meal [73]. In this study, the physical activities of the employees in the intervention worksites were greatly improved after a 12-month intervention, which should play an important role in preventing weight gain, although no significant weight loss was observed. This failure to find a weight change associated with the intervention may be partly due to no improvements occurring in weight-related dietary behaviors such as the consumption of fried food and sweetened beverages. Another possible reason might be attributed to the intervention intensity. Prior studies with weight loss observed showed that the interventions with higher intensity approaches were generally more effective than those with lower intensity approaches [74,75]. However, such high-intensive approaches were both more expensive and had a lower participation rate. In this study, although the lectures and activities mainly took place during working hours and at the workplace, some participants did not participate or did not complete all contacts with the reason given of a lack of time [76].

In addition to weight control, the improvement in dietary habits is another important hypothesis of this study, and was partly supported. Fruit and vegetable consumption was increased at the intervention worksites compared to the control worksites after a 12-month intervention, which contributes to weight loss in a long run. Prior research showed that, compared with those consuming less than 362 g of fruits and vegetables per day, people who consumed more than 698 g of fruits and vegetables per day were 74% likely to achieve an average weight loss of 3.4 kg or more within 10 years [77]. This shows that salient weight loss may lag behind the improvement in dietary behaviors, which is consistent with the hypothesis of this study. Moreover, for employees who are not overweight or obese, the benefits from the improvement in dietary behavior may be no less than, or even more important than, weight control. Studies have shown that lifestyle improvement, such as an increase in vegetable and fruit consumption, could still reduce the risk of obesity-related chronic diseases, even if there was no significant weight loss [78,79]. Intervention measures in our study, including the application of a WeChat official account, environmental improvement and EAB, played another important role in the improvement in dietary behaviors. Just as was mentioned in the prior literature, the use of modern communication tools could greatly improve participants’ compliance, and help achieve expected intervention effects [80,81,82]. In addition, creating a positive food environment at worksites can greatly increase employees’ access to a healthy diet, which can help employees develop and maintain healthy dietary behaviors [83,84]. In this study, based on the actual situation of each workplace, the healthy food of the workplace’s convenience stores was sold at a discount, which increased employees’ willingness to buy healthy food. Previous studies showed that when the price of healthy food is decreased by 25% and 50%, respectively, sales increase by 39% and 93%, respectively [85]. However, we also noticed that both the consumption of fried foods and sweet food and the frequency of eating out and at fast-food restaurants did not decrease as expected. One of the reasons may be that no intervention measures for canteens were conducted due to the lack of canteens at the worksites of this study. Fast food and takeout has become more and more popular among employees at the workplace in China, owing to their convenience and low cost. Fast food is usually supplied in large portions of low cost, high sugar and high-fat foods [86,87,88], which has been an obstacle to employees’ dietary intervention. A notable longitudinal study (during 2000–2009) of over 9000 Chinese adults from nine provinces in China revealed a positive association between fast food and central adiposity [89]. It might be a good strategy region wide to promote healthy food in a fast food or takeout context, which will be further explored in our future research.

Aside from the improvement in dietary behaviors, additional improvements occurred in employees’ physical activities of daily steps and self-efficacy in the intervention worksites compared to those in the control worksites. Walking is the most convenient and effective way to increase physical activity at the workplace, which is helpful to reduce weight and diastolic blood pressure [79]. Average daily steps of a typical 7 days were used to evaluate employees’ physical activities in the present study. The results showed that the daily steps of the control group decreased compared with those at the baseline, which was also reported in previous studies [90]. This may be a result of the increasing work pressure and longer working hours of employees in modern society. On the other hand, the daily steps of the intervention worksites did not increase significantly. In this study, the advocacy of using stairs instead of elevators by posters, the application of WeChat subscription as a convenient tool for goal setting and self-monitoring, the operation of various sports teams and economic stimulation effectively facilitated the maintenance of and improvement in employees’ physical activities. Studies have suggested that the main reason why employees chose an unhealthy lifestyle may not be laziness or lack of willpower, but a shortage of environmental support or access to healthy alternatives [91,92]. As indicated above, this study inspired employees to take stairs instead of elevators by posters and signs, creating a positive environment related to more physical activities at worksites. In addition to improving employees’ health awareness, it is more important to provide employees with alternative health behaviors. The “Exercise Challenge” based on a WeChat subscription helped employees set goals in stages, track data and rank the team, and then create an atmosphere of mutual motivation. The positive effect of spiritual and economic motivation on physical activity has been reported by several studies [93,94,95,96]. Besides the increase in daily steps, the proportion of employees with high exercise self-efficacy in the intervention worksites was also higher than that in the control worksites. The improvement in self-efficacy not only promoted the willingness to participate in physical activities, but also played an important role in maintaining physical activities [97]. However, we also noticed that no significant improvements were observed in the Godin index, the proportion of “exercise leading to sweating” and “walking days per week”. It is possible that the increase in daily steps in the intervention worksites was based on the accumulation of multiple times per day but shorter walks each time, which does not meet the time requirement of the Godin scale. However, using fragmented time to exercise in limited working hours is an effective and feasible way for sedentary employees to improve physical activity, which is also the characteristic of the intervention at worksites.

In recent years, the Chinese government has recognized the importance and urgency of overweight and obesity intervention at workplaces. Policies at the national level such as “Opinions on the Implementation of Healthy China Action” and “Outline of healthy China 2030” have been issued. However, worksite-based weight and weight-related lifestyle interventions in China are still relatively rare. Our findings suggest that implementation of the worksite-based lifestyle intervention on employees’ obesity control and prevention developed by this study could be beneficial. 

This study has some limitations. First, the representativeness of our study might be compromised due to modest sample size and a total response rate of 40.63%. Second, there was a small number of dropouts due to resignation, pregnancy or retirement after the intervention. The intention-to-treat analysis was adopted to reduce the information bias caused by dropouts. Third, neither the employees nor the researchers were blinded to the intervention assignment because of salient differences in procedures between intervention worksites and control worksites; however, there was no evidence that employees in the intervention worksites were more likely to report positive outcomes than those in the control worksites and vice versa. Our data collectors and analysts were blind to group assignment.

In summary, this group-randomized experimental study found that, after a 12-month worksite-based intervention to employees, improvements in lifestyle behaviors such as fruit and vegetable consumption, the frequency of sweetened beverage consumption, daily steps, and self-efficacy to change physical activity were found to be significantly different in the intervention worksites compared with the control worksites. The worksite-based lifestyle intervention on employees’ obesity control and prevention developed in this study is feasible and effective. Long-term intervention with larger samples could be further examined [98]. It is very important for worksites in China to develop and disseminate effective worksite-based weight intervention projects for obesity control or prevention, due to a large number of employees with unhealthy obesity-related behaviors.

## 5. Conclusions

The worksites-based lifestyle intervention project for obesity control and prevention improved several employees’ dietary behaviors and physical activities at worksites in China in a short time. Long-term intervention with larger samples in more worksites should be further examined.

## Figures and Tables

**Table 1:** Characteristics of the study participants at the baseline.

Characteristics		Intervention (N = 216)	Control (N = 172)	*p*-Value
Age (years)		30.83 ± 5.57	34.61 ± 9.82	<0.01 **
Gender	Male	143 (66.2)	100 (58.1)	0.10
	Female	73 (33.8)	72 (41.9)	
Marital status	Single or divorced	79 (36.6)	45 (26.2)	0.05
	Married or cohabiting	137 (63.4)	127 (73.8)	
Educational level	Under Junior college	5 (2.3)	37 (21.5)	<0.01 **
	Junior college	46 (21.3)	55 (32.0)	
	College or above	165 (76.4)	80 (46.5)	
Household incomes per capita (Yuan, USD 1 = 6.7 Yuan)	<24,000	29 (13.6)	21 (12.5)	0.87
	≥24,000, <56,000	49 (22.9)	42 (25.0)	
	≥56,000	136 (63.6)	105 (62.5)	

Data are presented as means ± SD or *n* (%); **: *p* < 0.01.

**Table 2:** Comparison of weight-related indicators between the intervention and control worksites from baseline to post-intervention.

	Intervention			Control			Adjusted Change between Groups, Mean (95%CI) ^☆^	Adjusted *p*-Value ^Δ^
	Baseline (N = 216)	Follow-Up (N = 159)	*p*-Value ^†^	Baseline (N = 172)	Follow-Up (N = 119)	*p*-Value ^†^		
BMI (kg/m^2^)	23.21 ± 3.71	22.95 ± 3.55	0.01 *	23.39 ± 3.15	23.33 ± 3.37	0.69	−0.11 (−0.82, 0.60)	0.76
WC (cm)	81.48 ± 11.28	80.82 ± 10.18	0.11	80.81 ± 9.59	80.60 ± 9.72	0.72	−0.89 (−2.63, 0.85)	0.31
HC (cm)	95.97 ± 6.44	95.28 ± 6.64	0.03 *	95.75 ± 6.16	95.53 ± 6.09	0.65	−0.11 (−1.34, 1.13)	0.87
WHR	0.85 ± 0.08	0.85 ± 0.07	0.98	0.84 ± 0.07	0.84 ± 0.06	0.88	−0.01 (−0.02, 0.002)	0.12
WHtR	0.49 ± 0.06	0.48 ± 0.05	0.01 *	0.49 ± 0.05	0.49 ± 0.05	0.47	−0.01 (−0.02, 0.01)	0.34

Data are presented as means ± SD. †: *p* values of within-group difference between baseline and post-intervention using paired *t*-tests; Δ: *p* values of between-group difference adjusted for the demographic characteristics. ☆: Changes in the indicators in intervention worksites from baseline to post-intervention compared with those in control worksites. *: *p* < 0.05.

**Table 3:** Comparison of eating-behavior changes between the intervention and control worksites from baseline to post-intervention.

	Intervention			Control			Adjusted Change between Groups, Mean (95%CI) ^☆^	Adjusted *p*-Value ^Δ^
	Baseline (N = 216)	Follow-Up (N = 159)	*p*-Value ^†^	Baseline (N = 172)	Follow-Up (N = 119)	*p*-Value ^†^		
Frequency of fried food per month	3.46 ± 4.34	2.73 ± 3.32	0.19	3.49 ± 5.58	3.24 ± 4.84	0.66	−0.11 (−1.04, 0.83)	0.82
Servings of fried food per month	4.91 ± 7.79	3.53 ± 5.39	0.15	3.90 ± 6.60	3.79 ± 7.07	0.89	0.81 (−1.17, 2.79)	0.42
Frequency of sweetened beverage per month	4.10 ± 5.37	3.13 ± 4.47	0.17	5.70 ± 11.41	4.20 ± 5.75	0.13	−1.81 (−0.52, −3.11)	<0.01 **
Servings of sweetened beverage per month	4.83 ± 6.69	4.04 ± 8.01	0.45	9.57 ± 35.80	4.63 ± 7.15	0.08	−2.44 (−5.68, 0.81)	0.14
Frequency of sweetened food per month	4.22 ± 6.02	3.29 ± 4.66	0.18	4.36 ± 7.06	3.27 ± 4.42	0.09	0.14 (−0.89, 1.18)	0.79
Servings of sweetened food per month	5.37 ± 10.59	3.55 ± 4.81	0.11	5.78 ± 15.77	3.53 ± 4.46	0.08	−0.25 (−2.00, 1.50)	0.78
Frequency of fruit per month	18.10 ± 16.12	18.47 ± 15.60	0.86	16.02 ± 14.41	15.63 ± 13.41	0.79	3.68 (1.25, 6.12)	<0.01 **
Servings of fruit per day	1.05 ± 1.22	0.93 ± 0.97	0.35	0.81 ± 1.03	0.77 ± 0.77	0.64	0.26 (0.44, 0.92)	<0.01 **
Frequency of vegetables per month	39.03 ± 22.97	44.49 ± 26.36	0.14	34.66 ± 24.92	41.25 ± 22.82	<0.01 **	5.66 (1.59, 9.74)	<0.01**
Servings of vegetables per day	1.94 ± 2.01	2.15 ± 1.78	0.46	1.53 ± 1.48	1.81 ± 1.51	0.06	0.53 (0.24, 0.82)	<0.01 **
Servings of vegetables/fruit per day	2.99 ± 2.72	3.10 ± 2.06	0.76	2.37 ± 1.98	2.61 ± 1.81	0.20	0.79 (0.43, 1.16)	<0.01 **
Frequency of eating out per month	3.80 ± 4.58	3.92 ± 4.19	0.85	3.50 ± 3.57	3.74 ± 4.21	0.57	0.53 (−0.21, 1.27)	0.16
Fast-food meals per month	1.12 ± 1.27	2.08 ± 2.89	<0.01 **	1.30 ± 1.69	2.09 ± 2.83	<0.01 **	0.05 (−0.43, 0.33)	0.79

Data are presented as means ± SD. †: *p* values of within-group difference between baseline and post-intervention using paired *t*-tests; Δ: *p* values of between-group difference adjusted for the demographic characteristics. ☆: Changes in the indicators in intervention worksites from baseline to post-intervention compared with those in control worksites. **: *p* < 0.01.

**Table 4:** Comparison of physical-activity changes between the intervention and control worksites from baseline to post-intervention.

		Intervention			Control			Adjusted Change between Groups, Mean/OR (95%CI) ^☆^	Adjusted *p*-Value ^Δ^
		Baseline (N = 216)	Follow-Up (N = 159)	*p*-Value ^†^	Baseline (N = 172)	Follow-Up (N = 119)	*p*-Value ^†^		
Godin index		22.67 ± 20.04	25.77 ± 27.98	0.18	24.86 ± 29.85	23.15 ± 19.48	0.88	1.04 (−3.59, 5.67)	0.66
Walking days per week		5.16 ± 2.12	4.84 ± 2.11	0.09	5.19 ± 2.30	5.21 ± 2.19	0.94	−0.34 (−0.75, 0.07)	0.10
Daily steps		6554.41 ± 3113.82	6919.76 ± 4412.09	0.36	6986.14 ± 2526.67	4994.83 ± 2226.66	<0.01 **	863.19 (161.42, 1564.97)	0.02 *
Exercise to sweating	Yes	117 (56.0)	98(63.6)	0.02 *	89 (54.3)	85(71.4)	0.03 *	0.68 (0.02, 20.57)	0.75
	No	92 (44.0)	56(36.4)		75 (45.7)	34(28.6)			
Exercise self-efficacy	High	85 (40.5)	53(34.9)	0.09	71 (42)	51(55.3)	0.58	1.91 (1.02,3.60)	0.04 *
	Low	125 (59.5)	99(65.1)		298(58)	63(44.7)			

Data are presented as means ± SD or *n* (%); †: *p* values of within-group difference between baseline and post-intervention using paired *t*-tests; Δ: *p* values of between-group difference adjusted for the demographic characteristics. ☆: Changes in the indicators in intervention worksites from baseline to post-intervention compared with those in control worksites. *: *p* < 0.05; **: *p* < 0.01.

## Data Availability

The data used to support the findings of this study are available from the corresponding author upon request.
